# Performance Evaluation of Fusing Protected Fingerprint Minutiae Templates on the Decision Level

**DOI:** 10.3390/s120505246

**Published:** 2012-04-26

**Authors:** Bian Yang, Christoph Busch, Koen de Groot, Haiyun Xu, Raymond N. J. Veldhuis

**Affiliations:** 1 Norwegian Biometric Laboratory, Gjøvik University College, Teknologivegen 22, N2802 Gjøvik, Norway; E-Mail: christoph.busch@hig.no; 2 Philips Research Eindhoven, High Tech Campus 5, 5656 AE Eindhoven, The Netherlands; E-Mail: koen.de.groot@philips.com; 3 Faculty EEMCS at University of Twente, P.O. Box 217, 7500 AE Enschede, The Netherlands; E-Mails: h.xu@utwente.nl (H.X.); r.n.j.veldhuis@utwente.nl (R.N.J.V.)

**Keywords:** decision level fusion, fingerprint, template protection, pseudonymous identifier, minutiae template, performance evaluation

## Abstract

In a biometric authentication system using protected templates, a pseudonymous identifier is the part of a protected template that can be directly compared. Each compared pair of pseudonymous identifiers results in a decision testing whether both identifiers are derived from the same biometric characteristic. Compared to an unprotected system, most existing biometric template protection methods cause to a certain extent degradation in biometric performance. Fusion is therefore a promising way to enhance the biometric performance in template-protected biometric systems. Compared to feature level fusion and score level fusion, decision level fusion has not only the least fusion complexity, but also the maximum interoperability across different biometric features, template protection and recognition algorithms, templates formats, and comparison score rules. However, performance improvement via decision level fusion is not obvious. It is influenced by both the dependency and the performance gap among the conducted tests for fusion. We investigate in this paper several fusion scenarios (multi-sample, multi-instance, multi-sensor, multi-algorithm, and their combinations) on the binary decision level, and evaluate their biometric performance and fusion efficiency on a multi-sensor fingerprint database with 71,994 samples.

## 1. Introduction

To achieve the purpose of biometric template protection, standard encryption mechanism, such as DES, AES, …, *etc.*, can be applied to convert the plaintext biometric templates to ciphertexts. However in this case, decryption is always required before comparison to tolerate the sample acquisition distortion of the fuzzy biometric signals. This demands an additional secure hardware environment for decryption and comparison. Moreover, standard encryption mechanisms become reversible when the secret key is available to the deciphering entity, such as in the system intrusion or insider attack scenarios. Unlike the standard encryption mechanisms which suffer from these drawbacks, many biometric template protection mechanisms have been proposed in recent years [1–10] to enhance the templates' security and privacy aspects by enabling renewable protected templates [11] and comparison thereof in a securely transformed domain. The security and privacy requirements [11] to these biometric template protection solutions are irreversibility (from the protected templates to original biometrics characteristics/features) and unlinkability (among diversified protected templates). Jain *et al.* categorized the popular biometric template protection algorithms into four approaches [12]—salting (biohashing or random projection [3]), non-invertible transform [4], key binding biometric cryptosystem [1,2], and key generating biometric cryptosystem [9–10]. Most existing template protection algorithms sacrifice their biometric performance in terms of recognition accuracy to achieve a higher security and privacy level. For biohashing and the non-invertible transform, diversification parameters (*i.e.*, projection vectors and transformation parameters) is insecure to be public [13] and thus have to be kept secret to guarantee the irreversibility and unlinkability [11] of the protected templates. Although an algorithm assuming public parameters can be secure (*i.e.*, [8]), it usually sacrifices biometric performance. Even in this secret parameters case, the performance may degrade because: (1) diversification parameters are random and thus not optimized to the input biometric feature and (2) both random projection and non-invertible transform are surjective functions and thus reduce the features' distinguishability especially in the case of short feature vectors. For the key binding biometric cryptosystem such as fuzzy commitment [1] and fuzzy vault [2], biometric performance is constrained to the feature robustness limited by the error correction capability. For the key generating biometric cryptosystem such as the secure sketches [9,10], the biometric performance can be constrained by the contradictive interaction between distinguishing ability and stability of the generated key due to the quantization essence of secure sketches. In addition, biometric template protection algorithms may suffer from the performance degradation due to the difficulty in absolute pre-alignment of plain biometric templates (such as in some hand-based modalities) while post-alignment can hardly be done in the protected domain.

On the other hand, the usability of biometric-enabled authentication systems demands well-preserved biometric performance from the protected templates compared to their plain counterparts. While template protection algorithms themselves are struggling in improving recognition performance, fusion based on multi-biometrics [14] provides another simple-but-effective way to achieve the same goal in a practical system. In general, fusion can be performed on four levels [14]: sample, feature, score, and decision levels; however, fusion on feature and score levels are difficult to realize for a given template protection system since these two levels usually interfere with the protection mechanism and algorithm design. Fusion at the sample level is however limited to the case that the original biometric sample instead of the derived biometric features (e.g., fingerprint minutiae) are used. We study the decision level fusion for protected templates in this paper because decision level fusion is independent of template protection algorithms and thus maximally adaptable to different template protection algorithms in addition to its high operating efficiency. Four fusion scenarios—multi-sample, multi-instance, multi-sensor, and multi-algorithm—and their combinations are tested under different sensors by several fingerprint minutiae based template protection algorithms developed in the research project TURBINE [15].

The remaining part of this work is organized as follows: Section 2 presents the concepts of pseudonymous identifiers used for biometric template protection and the tested template protection algorithms in this work; Section 3 provides background information of this performance evaluation work including the tested database, fusion scenarios, and testing settings; Section 4 presents the biometric performance testing results under different fusion scenarios; Section 5 gives a brief evaluation and analysis over the performance testing results. Section 6 concludes this work.

## 2. Fingerprint Minutiae Templates Protection Algorithms under Testing

### 2.1. Reference Architecture for Biometric Template Protection

**Figure 1.**
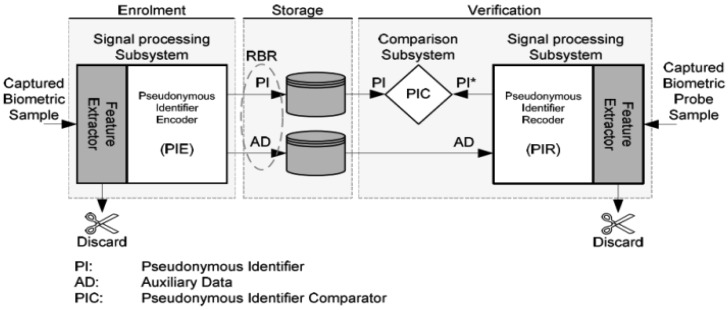
Reference architecture of template protection defined in [11].

A reference architecture (shown in Figure 1) was defined in [11], which can be used to analyze a biometric template protection mechanism's components in terms of functionality. In this reference architecture, a protected template (a.k.a reversible biometric reference (RBR) in Figure 1) consists of two parts—Pseudonymous Identifier (PI) and Auxiliary Data (AD).

A Pseudonymous identifier (PI) derived from biometric features is defined in [11] as “part of a renewable biometric reference that represents an individual or data subject within a certain domain by means of a protected identity that can be verified by means of a captured biometric sample and the auxiliary data (if any)”. The Auxiliary Data (AD) is defined in [11] as “subject-dependent data that is part of a renewable biometric reference and may be required to reconstruct pseudonymous identifiers during verification or for verification in general”. Simply to say, for a typical biometric template protection algorithm, PI is used as an identifier to a subject and for direct comparisons; AD is used for PI recoding or new PIs' reconstruction (*i.e.*, diversification). PI and AD can be separated logically or even physically in a practical system. Existing template protection mechanisms can be well mapped into this reference architecture (seen in Table D.1 in Annex D of [11]).

A RBR is required to have enough irreversibility and unlinkability [11] to guarantee the security and privacy of the original biometric characteristics/features. The irreversibility and unlinkability requirements are strictly imposed on PI which should be deemed as public data stored in a database. Depending on the application requirements and template protection algorithms' devise, AD can be public (e.g., fuzzy schemes [1,2,6] and dynamic random projection [8]), semi-public (*i.e.*, publishing AD will cause security level degradation, e.g., cancelable biometrics [4], random projection [3], and Biotoken scheme [5]), or must be kept secret (e.g., the case using standard encryption keys as AD). Since authentication of individuals is based on comparing PIs, decision level fusion is applied on individual PI comparison decisions in this work.

### 2.2. The Template Protection Algorithms under Testing

The minutiae feature is a standardized fingerprint feature that is widely adopted by existing fingerprint recognition systems. Template protection algorithms developed for minutiae features can thus be applied on any existing minutiae template based fingerprint recognition systems. In the TUBIRNE project, three fingerprint minutiae based template protection algorithms [6–8] were proposed by the project partners which all passed the security and privacy analysis [16] conducted by an independent project partner with cryptographic and cryptanalysis expertise. All algorithms were intended to meet the biometric performance target FRR ≤ 1% at FAR = 0.1%, which is more security-oriented than convenience-oriented, on a sequestered (publicly accessible but limited to on-premise use with dissemination prohibited by the Norwegian Data Privacy Agency) fingerprint database GUC100 [17]. The three algorithms generate a protected template from a plain ISO standard [18] conformed minutiae template each in a different way and result in different data syntax and formats for both PI and AD. The basic principle for each of the individual algorithms is briefly described as follows.

Algorithm 1 (Spectral minutiae based fuzzy commitment): its feature extraction was based on [6] which uses the spectral minutiae technique to obtain a stable and fixed-length binary vector from a fingerprint sample and subsequently performs a fuzzy commitment operation [1] to bind a randomly generated secret S with the binary representation which yields AD. The hashed secret S serves as PI. The fuzzy commitment scheme instead of the commonly-used fuzzy vault scheme was selected as the template protection method in this algorithm because fuzzy commitment can clearly separate the feature extraction step from the template protection step. This separation has two merits: first, the feature extraction method [6] can be improved in performance independently without causing too much negative impact on security; second, the security analysis [19] and enhancement [20] tasks can directly follow the information-theoretical way over the fuzzy commitment scheme working on fixed-length binary codes in a feature-agnostic way. Comparatively, fuzzy vault is increasingly criticized due to its non-separability in its feature extraction (simply minutiae coordinates) and the protection method (secret hiding by chaff points), which makes the linkability attack [21] (same minutiae coordinates among diversified templates), the substitution attack [21] (hiding imposter secrets as chaff points in the same vault), and also the brute-force attack [22] feasible.

Algorithm 2 (Minutiae vicinity based distance binarization): it was based on [7] which uses *N* randomly-generated minutiae vicinities (as AD) to measure their distance from a fingerprint vicinity and in this way *N* bits are generated as PI. External security protocols [23] are employed to guarantee the confidentiality of AD and thus the security of the template protection scheme as a whole.

Algorithm 3 (Minutiae vicinity based dynamic random projection): it was based on [8] which uses input-dependent random vectors in a random vectors group (thus the whole group can be public as AD under security analysis) to project a real-value minutiae vicinity vector constructed from neighboring minutiae relationship, which results in a binary sequence as PI. The security achieved by this algorithm is dependent on the computational complexity to find the genuine pre-image of a protected template.

The above-mentioned three developed algorithms in the TURBINE project represent three types of ideas for biometric template protection: (1) binary secret binding and release realized by fuzzy schemes (Algorithm 1) with binary and fixed-length PI; (2) hybrid (software + hardware) scheme (Algorithm 2) with binary and varied-length PI; and (3) irreversible transformation (Algorithm 3) with binary and varied-length PI. More information including biometric performance and security analysis of these three algorithms can be found in the references given in the above algorithm descriptions and the reference [16]. A quick look at their biometric performance can be found at the “without fusion” case in Figure 3 and Table 3 in Section 4.

Note that in the following sections, due to project partners' request, the above three algorithms are **anonymously flagged** as A1, A2 and A3, *i.e.*, A*_i_* (*i* = 1, 2, 3) does not necessarily denotes Algorithm *i* (*i* = 1, 2, 3) in the above. But throughout all the testing results, the three algorithms' flags A*_i_* (*i* = 1, 2, 3) don't change.

## 3. Decision Level Fusion Settings under Testing

Due to the challenging quality of the collected fingerprint samples in the GUC100 database, none of above-mentioned individual algorithms proposed by TURBINE met the performance target of the project. Hence, decision level fusion was proposed as a fallback plan to improve the recognition performance which finally achieved the goal. Decision level fusion was considered in this case just because the three algorithms have different characteristics and also different score and threshold setting mechanisms. It is also highly efficient to implement and configure in performance testing.

### 3.1. Rationale for Decision Level Fusion

Although score-level fusion is preferred in many multi-modality biometrics [24], it was not adopted in TURBINE because (1) it is not flexible enough to plug-in future algorithms for testing if their score and threshold settings are different and even incompatible to existing scoring systems (e.g., for fuzzy schemes [1,2] using mathematical hashing, only a binary decision thus a binary score can be obtained); (2) even score normalization schemes can be used to align the score dynamic range, they will inevitably cause information loss compared to raw scores [24]. Decision level fusion, however, does not suffer from such inconveniences and therefore was adopted in TURBINE.

Note that the argument that decision level fusion can improve the biometric performance does not always hold but strongly depends on the assumption of independence and marginal performance gap among the elements for fusion [25]. For example, using the fusion rule OR will increase the False Acceptance Rate (FAR) and at the same time decrease the False Rejection Rate (FRR); and using the fusion rule AND will have the opposite effect [this is easier to see from the Equations (1–4) in Section 3.3 under the case that the two elements for fusion are independent]. It is not necessary in theory that we can always obtain improve biometric performance through decision level fusion. The major factor is the performance gap between the two fusion elements. The fusion result could be worse than the one resulted from using the stronger element alone if the performance gap is distinct [25]. Another factor is the independence between the two elements for fusion–obviously dependency among fusion elements will reduce the information difference that can be exploited for performance improvement.

In TURBINE experiments, we observed some performance gaps among sensors and algorithms, but not noticeably among samples and instances. Whether these performance gaps will degrade the performance improving effort by applying fusion will be investigated in the experimental section. On the other hand, we assume there is dependence among the decisions obtained from different samples, sensors, and algorithms; and the dependence could even be among different instances from the same subject. To which degree such dependence among elements for fusion will degrade the performance improving effort by applying fusion will also be investigated. We describe different fusion scenarios and testing settings in the following sub-section.

### 3.2. GUC100 Database for Testing

**Figure 2.**
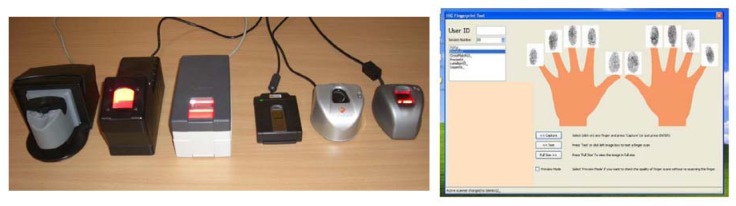
**Left**: fingerprint sensors used to collect data for GUC100 (from left to right): TST BiRD 3, L-1 DFR 2100, Cross Match L SCAN 100, Precise 250 MC, Lumidigm V 100 and Sagem MorphoSmart; **Right**: interface of the data collection program.

The GUC100 fingerprint database contains fingerprint samples collected in Norway in the winter-spring season of 2008. The samples are challenging in quality due to the cold and dry Scandinavian weather conditions, even though the collection took place inside a standard office room. This database was predominately used for testing purposes in the course of the TURBINE project and is also freely available to other researchers provided that testing is conducted at the GUC (Gjøvik University College) premises. It is a multi-sensor fingerprint database which had been created for independent and in-house performance and interoperability testing of third party algorithms. The samples were collected by six different fingerprint sensors, namely the TST BiRD 3, L-1 DFR 2100, Cross Match L SCAN 100, Precise 250 MC, Lumidigm V 100 and Sagem MorphoSmart (as shown in Figure 2 from left to right). Over several months, fingerprint samples from 12 sessions of all 10 fingers from 100 subjects (aged from teens to seniors with both genders balanced) on all six sensors were collected. This should result in 72,000 fingerprint images, but in practice only 71,994 valid images were recorded finally (the other six images are either from wrong fingers or are duplicates caused by software bugs and were thus removed).

### 3.3. Fusion Scenarios

Decision level fusion can be done in five scenarios [14]: multi-presentation, multi-sensor, multi-instance, multi-algorithm, and multi-modality. In our tests, only one sensor is used for each presentation of a finger, so the multi-presentation scenario translates to the multi-sample scenario with samples from different sessions. Also note that multi-instance in [14] and in this paper means multiple instances of the same type of biometric modality, e.g., different fingers, instead of multiple samples as done in [26]. Since only the fingerprint modality is concerned, we test the former four scenarios (multi-presentation, multi-sensor, multi-instance, and multi-algorithm) to improve the recognition performance of the pseudonymous identifier based protected fingerprint templates. In our experiments, there were in total eight fusion scenarios which were divided in three fusion layers tests as follows:

Single-layer fusion
  (1) Multi-sample fusion (*i.e.*, multi-presentation);
  (2) Multi-algorithm fusion;
  (3) Multi-sensor fusion;
  (4) Multi-instance fusion;
Bi-layer fusion
  (5) Multi-sample-algorithm fusion;
  (6) Multi-sample-sensor fusion;
  (7) Multi-sample-instance fusion;
Tri-layer fusion
  (8) Multi-sample-instance-algorithm fusion.

For the bi- and tri-layer fusion scenarios, we use the multi-sample fusion scenario as the basic layer because multi-sample is the easiest and also the most practically convenient scenario to realize fusion in a typical single-sensor-and-single-algorithm fingerprint recognition system—just to continuously capture multiple samples from one time of finger probing while asking the subject to slightly move the finger or modify the finger's contacting surface.

In our experiments, in light of testing efficiency, only binary decisions obtained from the left index finger (assumed to generating relatively good samples in quality) samples from 99 subjects (excluding one subject without full records) in 12 sessions are included for the first three fusion tests (multi-sample, multi-sensor, and multi-algorithm) and the right index finger is also used for the fourth fusion test (multi-instance). For the multi-sample fusion case, only binary decisions obtained from the a certain fingerprint sensor S and a minutiae extractor selected from a certain project partner have been fused, since in general best performances were observed from this sensor/minutiae-extractor combination. For the multi-sensor fusion, the three sensors L-1 DFR 2100, Precise 250 MC, and Sagem MorphoSmart in cooperation with the selected minutiae extractor are adopted. In this sensor fusion scenario, only the best biometric template protection algorithm A1 was employed for testing since in general best performances were observed from this algorithm/minutiae-extractor combination. Concerning both multi-algorithm fusion and multi-instance fusion, the sensor S and the selected minutiae extractor are used. To obtain the genuine scores for each subject, each sample out of the 12 samples (from 12 sessions) of the same finger was compared against the remaining 11 samples. In this way totally 12 × 11 × 99 = 13,068 genuine decisions were obtained. Each sample out of the 12 samples of the *N*^th^ subject (*N* = 1,2,…,98) was compared against the same-session samples out of all the other 99-*N* ({*N* + 1, *N* + 2,…,99}^th^) subjects. Note that the last subject (No. 99) is only contributing to the genuine decisions but not to the imposter decisions. In this way, Σ^98^_i = 1_12*i* = 58212 impostor scores were obtained.

To investigate decision level fusion's baseline effectiveness on performance improvement, we test only the simplest fusion rules such as AND and OR. In general, the decision OR rule may reduce the False Rejection Rate (FRR) but increases the False Acceptance Rate (FAR), whereas the decision AND rule may reduce the FAR but increases the FRR. This will be absolutely true if all the biometric tests are independent [25] and in this case the combined probability of False Reject *p*(FR) and False Accept *p*(FA) will be as follows using OR:

(1) $$p\left(\text{FR}\right)=p_{1}\left(\text{FR}\right)p_{2}\left(\text{FR}\right)$$

(2) $$p\left(\text{FA}\right)=p_{1}\left(\text{FA}\right)+p_{2}\left(\text{FA}\right)-p_{1}\left(\text{FA}\right)p_{2}\left(\text{FA}\right)$$

where *p*_1_ and *p*_2_ correspond to two independent biometric tests; and the case using AND:

(3) $$p\left(\text{FA}\right)=p_{1}\left(\text{FA}\right)p_{2}\left(\text{FA}\right)$$

(4) $$p\left(\text{FR}\right)=p_{1}\left(\text{FR}\right)+p_{2}\left(\text{FR}\right)-p_{1}\left(\text{FR}\right)p_{2}\left(\text{FR}\right)$$

In our tests, the OR-rule is adopted because in the TURBINE project a FAR = 0.1% was rigidly required to assure the security of the biometric authentication system. Under this context, the performance operation points in the Detection Error Trade-off (DET) curve before fusion are mainly distributed in the FAR range < 0.1% for all the three algorithms and thus OR is suitable to approach the performance target FAR = 0.1% while greatly reducing FRR. Although the independence condition may not be well satisfied in the fusion scenarios, we still expect the effect of reduced FRR brought by OR. The specific settings for the different fusion scenarios are given in the following sub-section.

### 3.4. Scenario Settings

There are both some common settings among all the scenarios and some special settings for each different scenario. Table 1 lists the common settings in all experiments and Table 2 differentiates the scenarios by those special settings. As mentioned in Section 2.2, due to project partners' request, the three template protection algorithms are **anonymously flagged** as A1, A2 and A3. Similarly, we **annonymize the three sensors** L-1 DFR 2100, Precise 250 MC, and Sagem MorphoSmart used in the experiments and flag them as S1, S2, and S3 (S is one of them) in the remaining part of this paper, but throughout all the testing results, the three sensors' flags S*_i_* (*i* = 1, 2, 3) don't change.

Besides the concise presentation of all the scenarios settings in the Table 1 and Table 2, we describe the settings for each scenario in a self-supporting way as follows:

#### (1) Multi-sample fusion

The studied left-index-finger samples were captured by the sensor S and processed into minutiae templates by the selected minutiae extractor. *M* binary decisions made from verifications of the *M* samples circularly-neighboring in the session order are fused. E.g., if we index the 11 binary decisions from genuine comparisons as d_1_, d_2_, …, d_11_, the 11 fused binary decisions are (Σ^*M*^_*i* = 1_d*_i_*) > 0, (Σ^*M*^^+1^_*i* = 2_d*_i_*) > 0, (Σ^*M*+2^_*i* = 3_d*_i_*) > 0, …, (Σ^11^_*i* = 10_d*_i_* + Σ^*M*-2^_*i* = 1_d*_i_*) > 0, and (Σ^11^_*i* = 11_d*_i_* + Σ^*M*-1^_*i* = 1_d*_i_*) > 0. All the three algorithms A1, A2, and A3 are for tests.

#### (2) Multi-algorithm fusion

The studied left-index-finger samples were captured by the sensor S and processed into minutiae templates by the selected minutiae extractor. By an arbitrary template protection algorithm, there are 13,068 genuine comparison decisions and 58,212 imposter comparison decisions that can be obtained. Every two algorithms among A1, A2, and A3 are fused to generate (13,068 + 58,212) binary decisions. Besides, all the 3 × (13,068 + 58,212) binary decisions obtained from all of A1, A2, and A3 are fused into (13,068 + 58,212) binary decisions.

#### (3) Multi-sensor fusion

The studied left-index-finger samples were processed into minutiae templates by the selected minutiae extractor. From data collected from an arbitrary sensor, there are 13,068 genuine comparison decisions and 58,212 imposter comparison decisions that can be obtained. Every two sensors among S1, S2, and S3 are fused to generate (13,068 + 58,212) binary decisions. All the 3 × (13,068 + 58,212) binary decisions from the three sensors S1, S2, and S3 are fused into (13,068 + 58,212) binary decisions. In this scenario, the template protection algorithm A1 is used for testing since it in general demonstrated the best biometric performance.

#### (4) Multi-instance fusion

The studied left-index-finger and right-index-finger samples were captured by the sensor S and processed into minutiae templates by the selected minutiae extractor. On an arbitrary finger, there are 13,068 genuine comparison decisions and 58,212 imposter comparison decisions that can be obtained. Both the (13,068 + 58,212) binary decisions from the left index finger and the (13,068 + 58,212) binary decisions from the right index finger are fused into (13,068 + 58,212) binary decisions. All three algorithms, A1, A2, and A3, are for tests.

#### (5) Multi-sample-algorithm fusion

This scenario further fuses the multi-sample fusion results obtained respectively from the three template protection algorithms A1, A2, and A3 into (13,068 + 58,212) binary decisions, *i.e.*, combination of scenario (1) and scenario (2).

#### (6) Multi-sample-sensor fusion

This scenario further fuses the multi-sample fusion results obtained respectively from the samples captured by the three sensors S1, S2, and S3 into (13,068 + 58,212) binary decisions, *i.e.*, a combination of scenario (1) and scenario (3).

#### (7) Multi-sample-instance fusion

This scenario further fuses the multi-sample fusion results obtained respectively from the left and the right index fingers into (13,068 + 58,212) binary decisions, *i.e.*, a combination of scenario (1) and scenario (4).

#### (8) Multi-sample-instance-algorithm fusion

This scenario further fuses the multi-sample-instance fusion results obtained respectively from the three template protection algorithms A1, A2, and A3 into (13,068 + 58,212) binary decisions, *i.e.*, a combination of (7) and (2).

## 4. Testing Results

The computational complexity in the cross-comparisons in performance evaluation phase of the TURBINE project was so large that it took months for four workstations to complete just a small scale (settings specified in Section 3.2) test under a limited range of parameters combinations. To achieve better testing efficiency but without loss of generality, all three template protection algorithms A1, A2, and A3 generate only four operational performance points distributed mainly in the FAR range ≤ 0.1% in the DET curves instead of a dense-populated DET curve before applying any fusion. Another reason that we chose to generate sparse-populated DET operation points is that the mathematical hash based algorithm—spectral minutiae based fuzzy commitment—only generate binary decisions from template comparisons and thus is not straightforwardly able to generate dense-populated operation points by thresholding the comparison scores like other two algorithms. As mentioned in Section 2 and 3, for the request from the project partners, we anonymize the three algorithms and the three sensors in the testing results and show only algorithm flags A1, A2, A3 and sensor flags S1, S2, S3 in all figures and discussions, *i.e.*, no clear links are indicated between the algorithms/sensors and their flags. The testing results from different fusion scenarios are given as follows:

### (1) Multi-sample fusion

**Figure 3.**
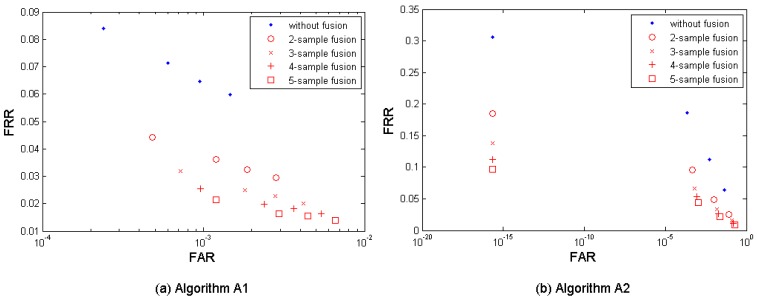

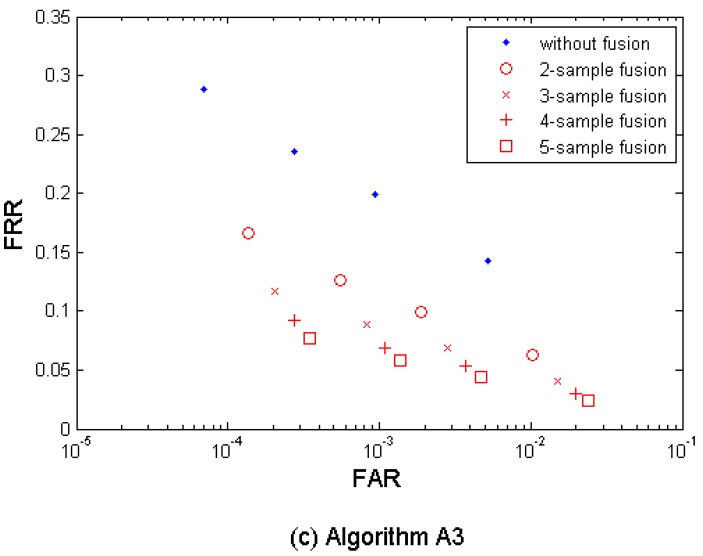
Multi-sample fusion results from the three anonymized biometric template protection algorithms A1, A2, and A3 developed in project TURBINE.

Figure 3(a–c) shows both the performance without fusion and the biometric performance of the multi-sample fusion by the algorithm A1, A2, and A3. Table 3 shows the performances which are closest to the target (FRR ≤ 1%, FAR = 0.1%) by the three algorithms. In the results, *M* = 2∼5 samples from neighboring sessions were used for fusion. It is clear to notice that the performance can be improved by increasing the number of samples used for fusion.

The algorithm A1 demonstrates the best performance but its performance point (FRR = 0.0256, FAR = 0.0010, while *M* = 4) closest to the target has not reached the target yet.

### (2) Multi-algorithm fusion

**Figure 4.**
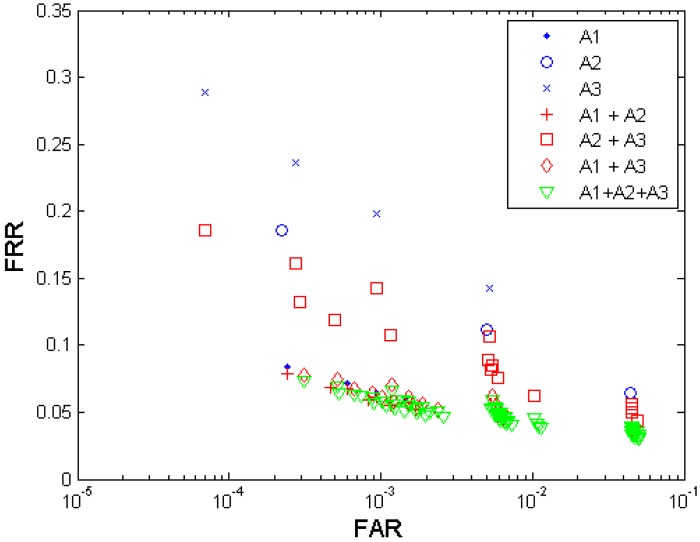
Multi-algorithm fusion results by the three anonymized biometric template protection algorithms A1, A2, and A3 developed in project TURBINE.

Figure 4 shows both the biometric performance without fusion and the performance with the multi-algorithm fusion from the algorithm A1, A2, and A3. Table 4 shows the performances which are closest to the target (FRR ≤ 1%, FAR = 0.1%) when fusing the three algorithms. Since each algorithm provides four performance points, any two algorithms' fusion generates 4 × 4 = 16 performance points, and all three algorithms' fusion generates 4 × 4 × 4 = 64 performance points. It is observed that the performance can be improved by fusion of decisions from different template protection algorithms. The algorithm A1 demonstrates better performance than A2 and A3, and even better than the fusion of A2 and A3. Compared to the performance achieved without fusion and that achieved by two-algorithm fusion, the fusion of all three algorithms resulted in best performance but its performance point (FRR = 0.0586, FAR = 0.0010) closest to the target has not reached the target yet.

### (3) Multi-sensor fusion

**Figure 5.**
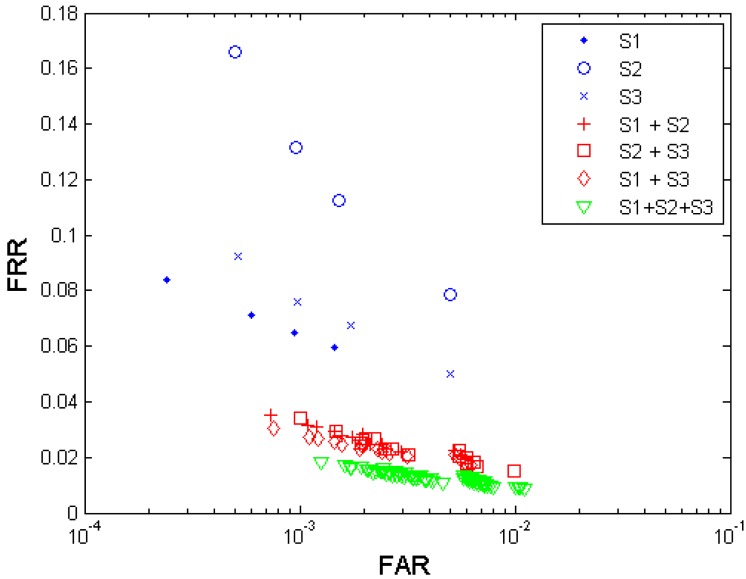
Multi-sensor fusion results from the three anonymized biometric sensors S1, S2, and S3 tested in project TURBINE, using algorithm A1.

Figure 5 shows both the biometric performance without fusion and the performance with the multi-sensor fusion from the three sensors S1, S2, and S3. Table 5 shows the performances which are closest to the target (FRR ≤ 1%, FAR = 0.1%) when fusing the three sensors.

Since from each sensor, four performance points were generated, any two sensors' fusion generates 4 × 4 = 16 performance points, and all three sensors' fusion generates 4 × 4 × 4 = 64 performance points. It is observed that the performance can be improved by fusion of decisions from different fingerprint sensors. Sensor S3 demonstrates a better performance compared to S1 and S2. The fusion of decisions from all three sensors resulted in the best performance but its performance point (FRR = 0.0340, FAR = 0.0010) closest to the target has not reached the target yet.

### (4) Multi-instance fusion

**Figure 6.**
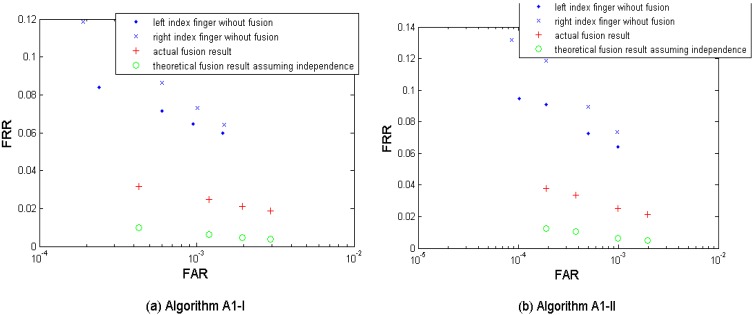

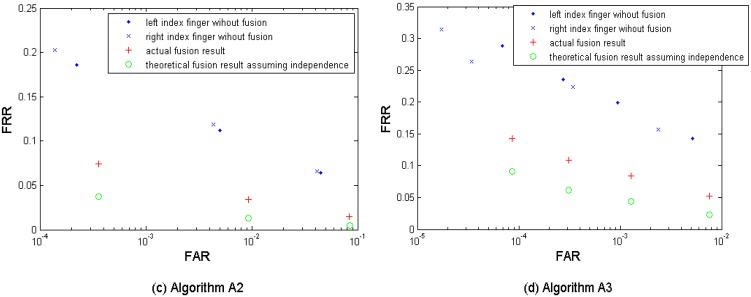
Multi-instance fusion results from the left and the right index fingers tested using the three algorithms A1 (Set I), A1 (Set II), A2, and A3 developed in project TURBINE.

Figures 6a–d show the biometric performance from the left and the right index fingers without fusion, the actual fusion results, and the theoretical fusion results estimated from Equations (1–2) assuming the independence of the two index fingers. Table 6 shows the performances which are closest to the target (FRR ≤ 1%, FAR = 0.1%) when fusing the two index fingers. The results were obtained by using the three algorithms A1, A2, and A3. In this scenario, we denote the corresponding thresholds for comparison scores by A1 to generate the original two groups of four performance points (for left and right index fingers respectively) as Set I, and then selected two new groups of thresholds as Set II which are larger (thus demanding a higher comparison score for a match) than Set I in order to generate two new groups of performance points with a lower FAR. The purpose of adding this second threshold group is mainly for keeping the performance points generated from the following bi- and tri-layer fusion scenarios remained in the range FAR ≤ 0.1%. It is clear to see that instance based fusion gives noticeable improvement in performance in all three algorithms. The fusion result using the algorithm A1 with the Set II thresholds resulted in best performance but its performance point (FRR = 0.0253, FAR = 0.0010) closest to the target has not reached the target yet. It is also interesting to note that there are conspicuous gaps in performance between the actual fusion results and the theoretical fusion results from all three template protection algorithms, which indicates dependency between the left and right index fingers. This dependency can be due to the sample quality, *i.e.*, both index fingers from the same subject are usually in similar physiological quality and we will verify this by experiments in Section 5.1, but is still worthy of an in-depth investigation in the future.

### (5) Multi-sample-algorithm fusion

**Figure 7.**
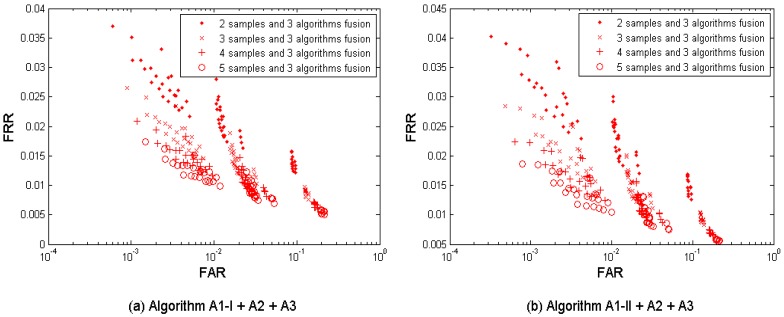
Multi-sample-algorithm fusion results from the tested three algorithms A1 (Set I), A1 (Set II), A2, and A3 developed in project TURBINE.

Figure 7(a-b) shows the multi-sample-algorithm fusion results from the three algorithms {A1-I, A2, A3} and {A1-II, A2, A3} respectively, with fusing *M* = 2, 3, 4, and 5 samples. Table 7 shows the performances which are closest to the target (FRR ≤ 1%, FAR = 0.1%) when fusing the three algorithms. Since each algorithm provides four performance points, the three algorithms result in 4 × 4 × 4 = 64 performance points. It is observed that the performance can be improved by increasing the number of samples used for fusion. This fusion test resulted in better performance than its single-layer fusion scenario (1) multi-sample and (2) multi-algorithm. But its performance point (FRR = 0.0222, FAR = 0.0010, while *M* = 4) closest to the target has not reached the target yet.

### (6) Multi-sample-sensor fusion

**Figure 8.**
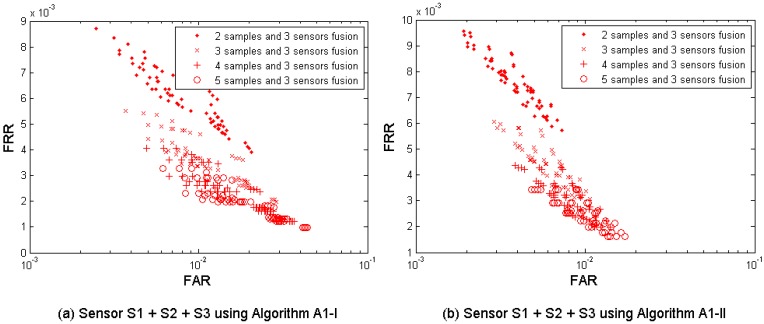
Multi-sample-sensor fusion results from the three sensors S1, S2 and S3, using the algorithm A1 (Set I) and A1 (Set II) developed in project TURBINE.

Figure 8(a–b) shows the multi-sample-sensor fusion results from all three sensors S1, S2, and S3, with fusing *M* = 2, 3, 4, and 5 samples. Table 8 shows the performances which are closest to the target (FRR ≤ 1%, FAR = 0.1%) when fusing the three sensors. Since from each sensor four performance points were generated, fusing three sensors results in 4 × 4 × 4 = 64 performance points. It is observed that the performance can be improved by fusion of decisions from different fingerprint sensors using multiple samples. This fusion test resulted in better performance than its single-layer fusion scenario (1) multi-sample and (3) multi-sensor in the concurrent FAR range (around FAR = 0.01). However, we note that all fusion results have FAR > 0.001 and are therefore outside the performance target range.

### (7) Multi-sample-instance fusion

**Figure 9.**
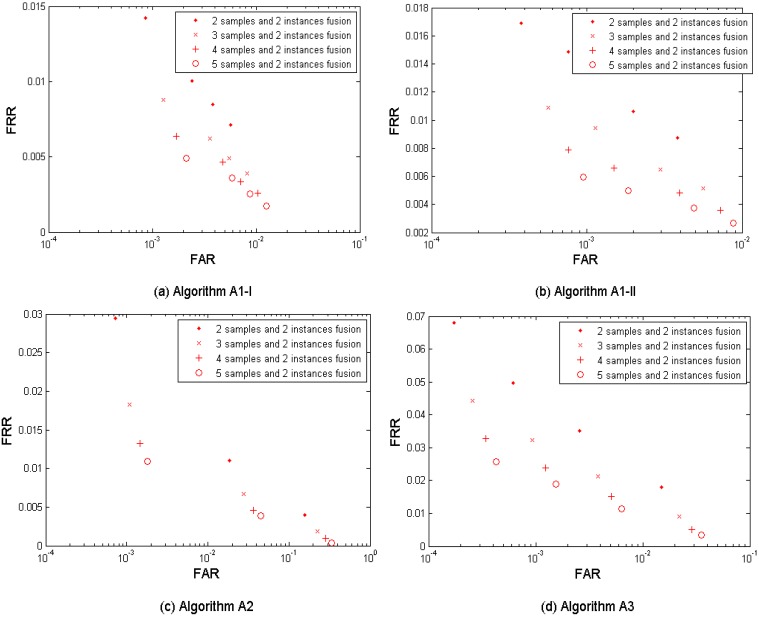
Multi-sample-instance fusion results from the left and the right index fingers, using the algorithms A1 (Set I), A1 (Set II), A2, and A3 developed in project TURBINE.

Figure 9(a–d) show the multi-sample-instance fusion results from the left and the right index fingers by fusing *M* = 2, 3, 4, and 5 samples and using the algorithms A1-I, A1-II, A2, and A3. Table 9 shows the performances which are closest to the target (FRR ≤ 1%, FAR = 0.1%) when fusing the two index fingers.

It is observed that the performance can be improved by increasing the number of samples used for fusion. This fusion test resulted in better performance than its single-layer fusion scenario (1) multi-sample and (4) multi-instance. It has two performance points (FRR = 0.0079, FAR = 0.0008, while *M* = 4 using A1-II; and FRR = 0.0060, FAR = 0.0009, while *M* = 5 using A1-II) that meet the target.

### (8) Multi-sample-instance-algorithm fusion

**Figure 10.**
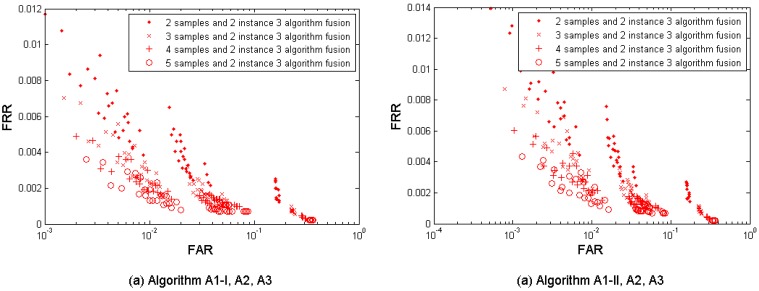
Multi-sample-instance-algorithm fusion results from the left and the right index fingers fusing the algorithms A1 (Set I), A1 (Set II), A2, and A3 developed in project TURBINE.

Figure 10(a–b) show the multi-sample-instance-algorithm fusion results from the left and the right index fingers by fusing *M* = 2, 3, 4, and 5 samples and fusing all three algorithms A1-I, A1-II, A2, and A3. Table 10 shows the performances which are closest to the target (FRR ≤ 1%, FAR = 0.1%) in this fusion scenario. Since each algorithm provides four performance points, fusing the three sensors results in 4 × 4 × 4 = 64 performance points. It is observed that the performance can be improved by increasing the number of samples used for fusion. This fusion test resulted in better performance than its single-layer fusion scenario (1) multi-sample, (2) multi-algorithm, and (4) multi-instance, but only marginally better (while *M* = 2 and 3) and no better (while *M* = 4 and 5) than the performance of the fusion scenario (7) multi-sample-instance.

It has two performance points (FRR = 0.0087, FAR = 0.0008, while *M* = 3 using A1-II; and FRR = 0.0060, FAR=0.0010, while *M* = 4 using A1-II) that meet the target. Comparison to the fusion scenario (7) multi-sample-instance shows that the performance improvement has been fully explored by the sample-and-instance fusion and the additional fused information from different algorithms did not gain noticeable improvement.

## 5. Performance Evaluation and Analysis

This section summarizes some experimental observations from our testing results. To fairly compare the fusion efficiency across different fusion scenarios, we evaluate the fusion efficiency via two criteria – efficiency per decision and efficiency per presentation.

### 5.1. General Experimental Observations

From the decision level fusion testing results, we can summarize the following experimental observations in a question (*Q*)/answer (*A*) way (note that all the answers apply only to our testing results and generalization to other cases should be carefully considered):

#### Observations on Performance Comparison

**Figure 11.**
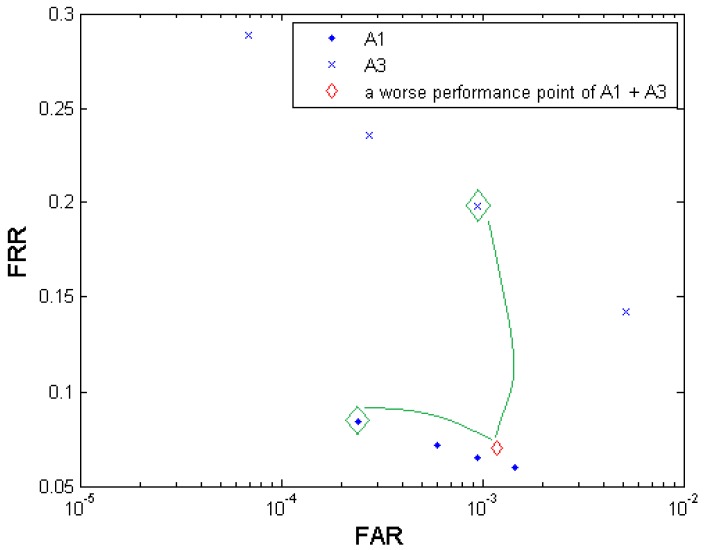
Example of performance degradation after decision-level fusion happened in the multi-algorithm scenario (the two big green diamonds denote the two operation points contributing to the small red diamond by fusion).

1. *Q*: Does decision-level fusion always result in a better biometric performance?
  *A*: In most cases decision-level fusion results in a better biometric performance if the two tested elements for fusion have similar biometric performance (small performance gap) on their own [25]. The amplitude of performance improvement depends on the dependency between the two elements. In our experiments, almost all the generated fusion results excel their primary elements. But we can still find some exceptional cases of performance degradation, e.g., in the multi-algorithm fusion test (Figure 4), the resultant fusion operation point (FAR = 0.0012; FRR = 0.0703), which is obviously worse than A1's performance in general, is generated from two primary operation points (FAR = 0.0002; FRR = 0.0840) in A1 and (FAR = 0.0009; FRR = 0.1985) in A3. In this case, the point (FAR = 0.0009; FRR = 0.1985) has a distinct performance gap from the point (FAR = 0.0002; FRR = 0.0840) in both FAR and FRR in the same time, which leads to performance degradation compared to the stronger biometric test A1. Figure 11 illustrates this performance degradation example. Fortunately in our experiments, such large-performance-gap tests were rarely observed.
2. *Q*: Is increasing the fusion layer effective in improving biometrics performance?
  *A*: Yes. Multi-layer (bi-layer and tri-layer) fusions in general perform better in recognition accuracy than single layer fusions if the multi-layer fusion has an increment based on the single-layer fusion, e.g., the multi-sample-instance fusion shows better performance than both the multi-sample fusion and the multi-instance fusion.
3. *Q*: Is increasing the number of decisions effective in improving biometrics performance?
  *A*: Yes. For a specific fusion scenario, increasing the number of decisions (*i.e.*, number of samples, algorithms, sensors, or instances) for fusion improves the recognition accuracy. But this gain in performance improvement is getting marginal with the increase of the number of decisions.
4. *Q*: What is the rank of the four cases (sample, algorithm, sensor, and instance) in effectiveness to achieve performance improvement?
  *A*: Instance is seemingly the most effective case to the recognition accuracy improvement, which can be attributed to their high independency among each other compared to that among samples, algorithms, and sensors. Followed in the order of effectiveness are sensor, sample, and algorithm.

#### Observations on Dependency among Elements for Fusion

**Figure 12.**
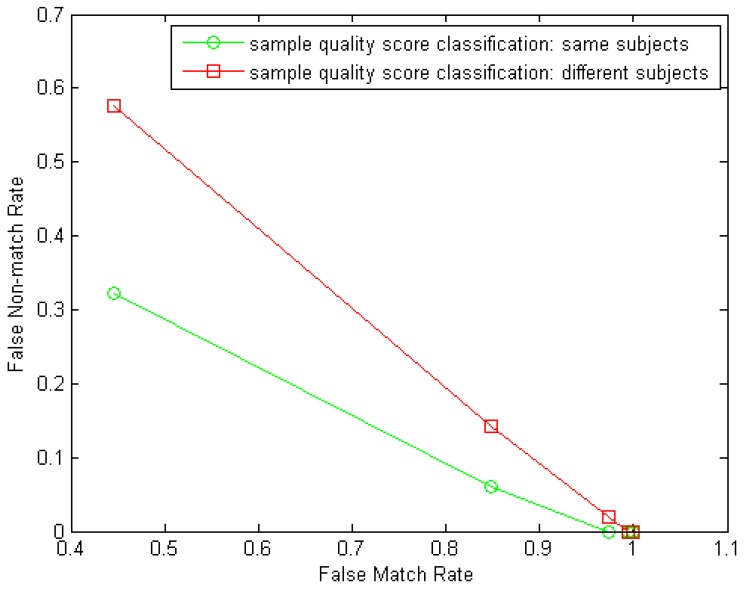
Example of two classification tests to verify the correlation in fingerprint images' quality from two same-subject index fingers. Samples are from one session of the GUC100 database. Classification test 1 (green curve): same-subject index finger samples are used to calculate genuine NFIQ score distances; classification test 2 (red curve): different-subject index finger samples are used to calculate “genuine” NFIQ score distances.

1. *Q*: Is there any dependency between the instances from the same subjects observed via fusion results?
  *A*: Instances, if being assumed of no distinct performance gap among each other, however, seems not completely independent among each other under the decision level fusion tests. We can see this clearly from Figure 6. This might be attributed to the correlation in fingers' physiological quality from the same subject. We investigated this correlation by comparing the NFIQ fingerprint quality scores [27] of the left-index-finger samples and the right-index-finger samples in our experiments–first, we form a genuine distance group by calculating the absolute values of the NFIQ score differences of the two same-subject index fingers from the 100 subjects and then form an imposter distance group by calculating the absolute values of NFIQ score differences of any two different-subject index fingers from the 100 subjects. Then classification of the two distance groups can show the correlation of the two instances from the same subject. In our test, on samples from 10 sessions in the GUC100 database, the average classification performance point closest to the Equal-Error-Rate (EER) (note that due to the narrow dynamic range of NFIQ scores (only 1∼5) an accurate EER was not able to obtain) achieves FMR = 0.5054 while FNMR = 0.3460. For comparison, we form another two distance groups for classification in which all distances are calculated from different subjects and this classification test achieves FMR = 0.5054 while FNMR = 0.4950 in average from 10 sessions. Now we can see the noticeable correlation in sample quality between the two same-subject instances from the former classification test. An example of the two classification results (DET curves) from one session in the GUC100 database is shown in Figure 12.
2. *Q*: Is there any dependency among the algorithms observed via fusion results?
  *A*: Algorithms, which themselves were supposed to be independent, may generate deeply-correlated decisions and/or have noticeable performance gaps among each other, which makes the fusion results not so impressive in our experiments as expected in recognition accuracy improvement compared to sample, sensor, and instance.
3. *Q*: Is there any supplementary information that can be exploited to improve the performance from multiple samples captured from same and multiple sensors which are assumed to be highly dependent?
  *A*: The decisions obtained from multiple samples captured from the same or different sensors are not completely redundant, thus enabling in our multi-sample fusion and the multi-sensor fusion experiments a gain in recognition accuracy.

Besides the above general observations, we found that only scenario (7) multi-sample-instance fusion and scenario (8) multi-sample-instance-algorithm fusion have some performance points that meet the recognition accuracy performance target for the project TURBINE. The best fusion result [FRR = 0.0060, FAR = 0.0009, while *M* = 5 using A1-II in the fusion scenario (7)] achieves around 91% reduction in FRR compared to the best performance without fusion (FRR = 0.0647, FAR = 0.0009, using A1-I).

### 5.2. Fusion Efficiency Comparisons

Considering the number of decisions and convenience of finger probing can vary in different fusion scenarios, we need to evaluate the fusion efficiency in addition to the effectiveness we observed from the testing results and discussed in Section 5.1. To compare the fusion efficiency of different fusion scenarios in a more precise way than the general evaluation given in Section 5.1, we propose two criteria—efficiency per decision and efficiency per presentation—to evaluate the fusion efficiency.

#### (1) Efficiency per decision

**Figure 13.**
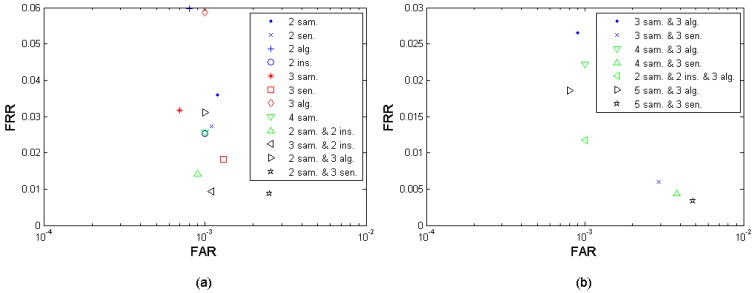
Fusion efficiency by “efficiency per decision”. (“sample”, “algorithm”, “sensor”, and “instance” are abbreviated as “sam.”, “alg.”, “sen.”, and “ins.”, respectively).

Efficiency per decision is defined as the performance achievable by fusing the same amount of decisions, *i.e.*, exploiting the same amount of fusion sources. Since the number of decisions as fusion sources are equivalent, the performance is more comparable. E.g., the 2-sample fusion, 2-sensor fusion, 2-algorithm fusion, and 2 instances fusion cases are comparable since they have the same amount of source decisions. Following the same logic, other examples can be that the 4-sample fusion is comparable to the 2-sample-and-2-instance fusion, and the 3-sample-and-2-instance fusion is comparable to the 2-sample-and-3-algorithm fusion. By this efficiency per decision criterion, we can compare the different fusion cases' efficiency when they have the same amount of source decisions. Figures 13 a–b present the fusion performance points that are closest to the performance target (FRR ≤ 1%, FAR = 0.1%) under different fusion scenarios, in which the points with the same amount of decision sources (marked in the same color) are comparable among each other. On the other hand, if two fusion cases demonstrate the similar performance, the one using fewer source decisions has the advantage in storage and operational/computational complexity. For example, the 2-instance fusion and the 4-sample fusion in the Figure 13a demonstrate roughly the same performance but the 2-instance fusion scenario excels under this efficiency per decision criterion. Table 11 translates the different fusion cases to their specific fusion scenarios settings defined in Section 3.4.

#### (2) Efficiency per presentation

**Figure 14.**
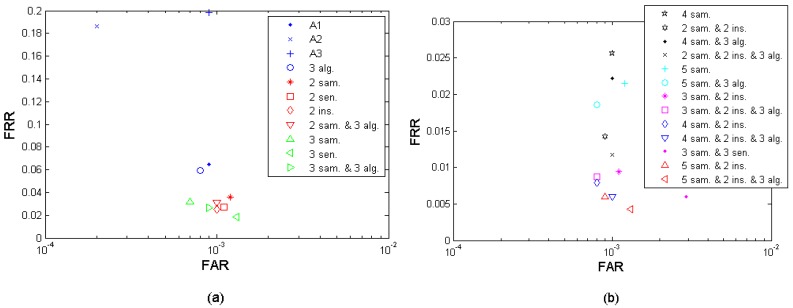
Fusion efficiency by “efficiency per presentation”. (“sample”, “algorithm”, “sensor”, and “instance” are abbreviated as “sam.”, “alg.”, “sen.”, and “ins.”, respectively)

Efficiency per presentation is defined as the performance achievable under the same amount of finger presentations (times of probing), which takes into account the convenience to the subjects. Since the effort required is almost the same under the same amount of finger presentations, the performance is more comparable. This efficiency criterion matters mainly on the multi-algorithm fusion since it needs only one finger presentation. Figure 14(a,b) presents the fusion performance points that are closest to the performance target (FRR ≤ 1%, FAR = 0.1%) under different fusion scenarios, in which the points with the same amount of finger presentation (marked in the same color) are comparable among each other. On the other hand, if two scenarios have the same amount of finger presentations, it is likely that the scenario which fuses more algorithms achieves a higher performance. For example, the 3-sample-and-2-instance-and-3-algorithm fusion and the 3-sample-and-2-instance fusion in the Figure 14(b) have the same amount of finger presentations but the former excels in performance. Table 12 translates the different fusion cases to their specific fusion scenarios settings defined in Section 3.4.

## 6. Conclusions

We presented in this paper an evaluation of decision level fusion results of fingerprint minutiae based pseudonymous identifiers generated by three biometric template protection algorithms developed in the European research project TURBINE. There are eight different fusion scenarios covering multiple samples, algorithms, sensors, instances, and their combinations in our tests. Distinct biometric performance improvements were observed by decision level fusion which verifies the hypothesis to apply fusion to improve the recognition accuracy performance. For fair comparisons of achieved performance improvement, two fusion efficiency criteria were proposed to evaluate the different scenarios' fusion efficiency.

Future work will investigate other fusion rules such as AND, layered, and cascaded fusion; and also the in-depth implications of performance improvement by the fusion rule OR and the dependency among elements for fusion. With regards to privacy, we assume that the template protection algorithms used are secure and privacy-enhanced. However, decision level fusion implies linkability among the protected templates generated out of different samples, sensors, instances, and algorithms from the same biometric characteristic (in the sample, sensor, and algorithm cases) and the same subject (in the instance case). Whether or not this fact influences the protected templates' security is quite algorithm-dependent, and therefore needs case-based security analysis.

## Figures and Tables

**Table 1.** Common settings for all fusion scenarios.

Algorithm	A1 (Annonymized flag)
Sensor	S (one of S1, S2, and S3)
Finger instance	Left index finger
Minutiae extractor	Selected one from one project partner
Number of genuine scores generated from each algorithm/sensor/finger	13,068
Number of imposter scores generated from each algorithm/sensor/finger	58,212

**Table 2.** Special settings for different fusion scenarios.

	**Algorithm**	**Sensor**	**Finger instance**	**Nr. of samples**
Multi-sample	A1, A2, A3	S	left index	2∼5
Multi-algorithm	A1 + A2, A2 + A3, A1 + A3, A1 + A2 + A3	S	left index	1
Multi-sensor	A1	S1 + S2, S2 + S3, S1 + S3, S1 + S2 + S3	left index	1
Multi-instance	A1, A2, A3	S	left index + right index	1
Multi-sample-algorithm	A1 + A2 + A3	S	left index	2∼5
Multi-sample-sensor	A1	S1 + S2 + S3	left index	2∼5
Multi-sample-instance	A1, A2, A3	S	left index + right index	2∼5
Multi-sample-instance-algorithm	A1 + A2 + A3	S	left index + right index	2∼5

**Table 3.** Best performance (operation points closest to the performance target FRR ≤ 0.01 @ FAR = 0.001)—multi-sample fusion.

**Number of samples used for fusion**	**Error rates**	**A1**	**A2**	**A3**
1 (without fusion)	FAR	0.0009	0.0002	0.0009
	FRR	0.0647	0.1860	0.1985

2	FAR	0.0012	0.0004	0.0005
	FRR	0.0360	0.0952	0.1267

3	FAR	0.0007	0.0007	0.0008
	FRR	0.0318	0.0667	0.0882

4	FAR	0.0010	0.0009	0.0011
	FRR	0.0256	0.0529	0.0690

5	FAR	0.0012	0.0011	0.0014
	FRR	0.0215	0.0439	0.0576

**Table 4.** Best performance (operation points closest to the performance target FRR ≤ 0.01 @ FAR = 0.001)—multi-algorithm fusion.

**Algorithm settings**	**A1 + A2**	**A2 + A3**	**A1 + A3**	**A1 + A2 + A3**
FAR	0.0008	0.0012	0.0010	0.0010
FRR	0.0597	0.1078	0.0614	0.0586

**Table 5.** Best performance (operation points closest to the performance target FRR ≤ 0.01 @ FAR = 0.001)—multi-sensor fusion.

**Sensor settings**	**S1 + S2**	**S2 + S3**	**S1 + S3**	**S1 + S2 + S3**
FAR	0.0011	0.0010	0.0011	0.0013
FRR	0.0315	0.0340	0.0273	0.0182

**Table 6.** Best performance (operation points closest to the performance target FRR ≤ 0.01 @ FAR = 0.001)—multi-instance (left and right index fingers) fusion.

**Algorithm**	**A1-I**	**A1-II**	**A2**	**A3**
FAR	0.0012	0.0010	0.0004	0.0013
FRR	0.0247	0.0253	0.0740	0.0835

**Table 7.** Best performance (operation points closest to the performance target FRR ≤ 0.01 @ FAR = 0.001)—multi-sample-algorithm fusion.

**Number of samples used for fusion**	**Error rates**	**A1-I +A2+A3**	**A1-II +A2+A3**
2	FAR	0.0010	0.0011
	FRR	0.0312	0.0317

3	FAR	0.0009	0.0011
	FRR	0.0265	0.0268

4	FAR	0.0012	0.0010
	FRR	0.0208	0.0222

5	FAR	0.0015	0.0008
	FRR	0.0175	0.0186

**Table 8.** Best performance (operation points closest to the performance target FRR ≤ 0.01 @ FAR = 0.001)—multi-sample-sensor fusion.

**Number of samples used for fusion**	**Error rates**	**S1 + S2 + S3 A1-I**	**S1 + S2 + S3 A1-II**
2	FAR	0.0025	0.0019
	FRR	0.0087	0.0094

3	FAR	0.0037	0.0029
	FRR	0.0055	0.0060

4	FAR	0.0049	0.0038
	FRR	0.0040	0.0044

5	FAR	0.0061	0.0048
	FRR	0.0033	0.0034

**Table 9.** Best performance (operation points closest to the performance target FRR ≤ 0.01 @ FAR = 0.001)—multi-sample-instance (left and right index fingers) fusion.

**Number of samples used for fusion**	**Error rates**	**A1-I**	**A1-II**	**A2**	**A3**
2	FAR	0.0009	0.0008	0.0007	0.0006
	FRR	0.0142	0.0149	0.0295	0.0497

3	FAR	0.0013	0.0011	0.0011	0.0009
	FRR	0.0088	0.0094	0.0183	0.0323

4	FAR	0.0017	0.0008	0.0014	0.0012
	FRR	0.0064	0.0079	0.0132	0.0239

5	FAR	0.0021	0.0009	0.0018	0.0015
	FRR	0.0049	0.0060	0.0109	0.0188

**Table 10.** Best performance (operation points closest to the performance target FRR ≤ 0.01 @ FAR = 0.001)—multi-sample-instance-algorithm fusion.

**Number of samples used for fusion**	**Error rates**	**A1-I +A2+A3**	**A1-II +A2+A3**
2	FAR	0.0010	0.0009
	FRR	0.0117	0.0123

3	FAR	0.0015	0.0008
	FRR	0.0070	0.0087

4	FAR	0.0020	0.0010
	FRR	0.0049	0.0060

5	FAR	0.0025	0.0013
	FRR	0.0036	0.0043

**Table 11.** Fusion scenario settings for fusion cases by “efficiency per decision”.

Decision amount for fusion	Fusion case	Scenario settings

Fusion from 2 decisions	2 samples	Multi-sample, *M* = 2, A1
	2 sensors	Multi-sensor, S1 + S3
	2 algorithms	Multi-algorithm, A1 + A2
	2 instances	Multi-instance, A1

Fusion from 3 decisions	3 samples	Multi-sample, *M* = 3, A1
	3 sensors	Multi-sensor, S1 + S2 + S3
	3 algorithms	Multi-algorithm, A1 + A2 + A3

Fusion from 4 decisions	4 samples	Multi-sample, *M* = 4, A1
	2 samples and 2 instances	Multi-sample-instance, *M* = 2, A1

Fusion from 6 decisions	3 samples and 2 instances	Multi-sample-instance, *M* = 3, A1
	2 samples and 3 algorithms	Multi-sample-algorithm, *M* = 2
	2 samples and 3 sensors	Multi-sample-sensor, *M* = 2

Fusion from 9 decisions	3 samples and 3 algorithms	Multi-sample-algorithm, *M* = 3
	3 samples and 3 sensors	Multi-sample-sensor, *M* = 3

Fusion from 12 decisions	4 samples and 3 algorithms	Multi-sample-algorithm, *M* = 4
	4 samples and 3 sensors	Multi-sample-sensor, *M* = 4
	2 samples and 2 instances and 3 algorithms	Multi-sample-instance-algorithm, *M* = 2

Fusion from 15 decisions	5 samples and 3 algorithms	Multi-sample-algorithm, *M* = 5
	5 samples and 3 sensors	Multi-sample-sensor, *M* = 5

**Table 12.** Fusion scenario settings for fusion cases by “efficiency per presentation”.

**Presentation amount for fusion**	**Fusion case**	**Scenario settings**

Fusion from single presentation	A1	Without fusion, A1
	A2	Without fusion, A2
	A3	Without fusion, A3
	3 algorithms	Multi-algorithm, A1 + A2 + A3

Fusion from 2 presentations	2 samples	Multi-sample, *M* = 2, A1
	2 sensors	Multi-sensor, S1 + S3
	2 instances	Multi-instance, A1
	2 samples and 3 algorithms	Multi-sample-algorithm, *M* = 2

Fusion from 3 presentations	3 samples	Multi-sample, *M* = 3, A1
	3 sensors	Multi-sensor, S1 + S2 + S3
	3 samples and 3 algorithms	Multi-sample-algorithm, *M* = 3

Fusion from 4 presentations	4 samples	Multi-sample, *M* = 4, A1
	2 sample and 2 instances	Multi-sample-instance, *M* = 2, A1
	4 samples and 3 algorithms	Multi-sample-algorithm, *M* = 4
	2 samples and 2 instances and 3 algorithms	Multi-sample-instance-algorithm, *M* = 2

Fusion from 5 presentations	5 samples	Multi-sample, *M* = 5, A1
	5 samples and 3 algorithms	Multi-sample-algorithm, *M* = 5

Fusion from 6 presentations	3 sample and 2 instances	Multi-sample-instance, *M* = 3, A1
	3 samples and 2 instances and 3 algorithms	Multi-sample-instance-algorithm, *M* = 3

Fusion from 8 presentations	4 sample and 2 instances	Multi-sample-instance, *M* = 4, A1
	4 samples and 2 instances and 3 algorithms	Multi-sample-instance-algorithm, *M* = 4

Fusion from 9 presentations	3 samples and 3 sensors	Multi-sample-sensor, *M* = 3

Fusion from 10 presentations	5 sample and 2 instances	Multi-sample-instance, *M* = 5, A1
	5 samples and 2 instances and 3 algorithms	Multi-sample-instance-algorithm, *M* = 5
